# Role of Intermolecular Forces on the Contact Angle of Vegetable Oil Droplets during the Cooling Process

**DOI:** 10.1155/2018/5283753

**Published:** 2018-07-03

**Authors:** Muhammad Akhlis Rizza, Widya Wijayanti, Nurkholis Hamidi, I. N. G. Wardana

**Affiliations:** ^1^Department of Mechanical Engineering, State Polytechnic of Malang, Malang, Indonesia; ^2^Department of Mechanical Engineering, Brawijaya University, Malang, Indonesia

## Abstract

This study aims to experimentally determine the role of intermolecular forces on the contact angle of vegetable oil droplets. Contact angles were recorded using a microscope and measured using digital software. The results show that the surface tension of vegetable oils is influenced by the London force between the electron clouds of molecules. The process of cooling increases vegetable oil contact angles, due to the decreased kinetic energy of constituent molecules and increased London force on the molecules. A decrease in temperature causes the surrounding water vapor to condense, which adheres to the droplet surface (due to the hydrophilic properties of molecules). Hydrogen bonds develop after moisture adheres to the surface. Further, water molecules on the droplet surface reduce the surface tension, because of hydrogen bonds between the molecules on the droplet surface and moisture. Hydrogen bonds among the molecules force water molecules to accumulate on the droplet surface, which suppresses the droplet surface; therefore the contact angle decreases.

## 1. Introduction

Wettability, statistical physics, and fluid dynamics have always been associated with the wetting phenomena of liquids on solids [1–4]. The application of wettability occurs in fouling phenomena, where fouling is defined as the accumulation of undesirable materials on a surface [5].

Fouling is a problem in processes that use a membrane. Fouling causes a significant decrease in fluxes by several types of dopants (impurities) such as adsorption, pore-clogging, and the aggregation of solutes on membrane surfaces. Most research on fouling has been on large deposits, such as pore closing by a layer of dirt. However, research on fouling due to small deposits, such as vegetable oil droplets, has been scarce [6]. Some research has found that processing edible oil can cause the fouling of fatty acids in a membrane, and these fatty acids are generally small. Moreover, fouling should be cleaned immediately, so that the quality of the product is not affected [7].

Certain forces must be overcome to enable the cleaning of impurities when fouling occurs: cohesive forces in impurity materials (deposits) and adhesion forces between the deposits and the surface. Researchers have suggested the need for improving cleanability as a solution for the problem of fouling [8].

The measurement of contact angles between solid surfaces and droplets is a procedure performed by researchers to evaluate the cleanability of droplets. Moreover, the contact angles between solid surfaces and droplets are the result of adhesion forces between liquids and solids [9].

In examining cleanability, several researchers tested the correlation of the thermodynamic model of adhesion forces between droplets and substrates, using experimentally measured adhesion forces [10]. Researchers also experimentally measured the adhesion forces occurring in some types of vegetable oils (olive oil, soybean oil, sunflower oil, and Vaseline oil) and some substrates (low-density polyethylene** (**LDPE), polyethylene terephthalate (PET), stainless steel, and glass). These were then compared to some thermodynamic models, and the results showed a strong correlation between the thermodynamic model of Young–Dupré and the experimental results.

Other studies regarding cleanability have been conducted, using a statistical testing method. Olive oil was dropped onto various coated substrates: ceramic coating, quasicrystalline coatings, polytetrafluoroethylene coating, and silicone coating. The substrates were then heated, and the results showed a relationship between decreased contact angles and increased temperatures [9].

Another experiment on cleanability proved that liquid metal dripped onto ceramics causes an increase in contact angles, with decreased temperatures. Surface roughness, porosity, and structural transformation of the substrate affected the increase in contact angles. Moreover, changes in droplet contact angles due to volume addition (or reduction) have also been investigated. Further, it has been proved that activation energy (due to interfacial changes in the area) affects contact angle changes [11].

Several studies have discussed changes in contact angles. However, it is difficult to find an explanation about the roles of intermolecular forces in contact angle changes, especially in long-chain molecules. Therefore, this study used three kinds of vegetable oil droplets to clarify the role of intermolecular forces of long carbon-chain molecules, especially the force that emerges from polar and nonpolar molecules. The selected vegetable oils were coconut oil, jatropha curcas oil, and sunflower oil. These vegetable oils were chosen because they contain different molecules. Most of the content of coconut oil is saturated fatty acid molecules, which are polar. Jatropha curcas oil contains monounsaturated fatty acids, and sunflower oil contains polyunsaturated fatty acids, both of which are lower in polarity.

## 2. Materials and Methods

**Figure 1:**
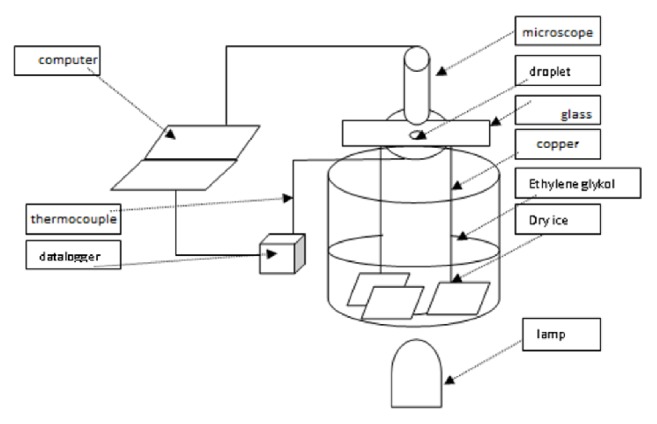
Experimental apparatus.

Droplets of the selected vegetable oils were dripped onto a glass surface, as shown schematically in Figure 1. The glass was attached to a sheet of copper, which functioned as a heat conductor from the droplet to the dry ice. Dry ice was soaked in ethylene glycol, so that the cooling process was even. The temperature measurements were carried out using a T-type thermocouple (via a datalogger), and the images were captured at 250x magnification using a Dino-Lite digital microscope. Imagej software was used to measure the contact angle.

The oils tested in this experiment were coconut, jatropha curcas, and sunflower, and their fatty acid compositions are tabulated in Tables 1, 2, and 3, respectively. As shown in the tables, coconut oil is composed mostly of lauric acid and jatropha curcas oil is composed mainly of oleic and linoleic acids, whereas sunflower oil is composed mainly of linoleic acid.

## 3. Results

**Figure 2:**
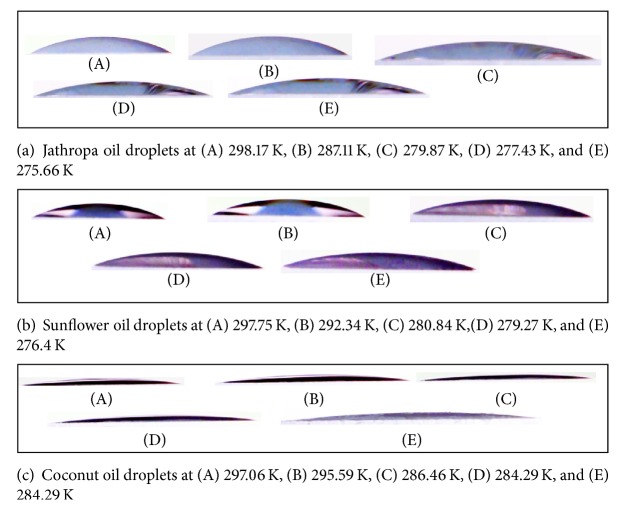


The results show that the cooling process affected the contact angles and the changes in contact angle occurred in all the samples.  Figure 2 shows the evolution of vegetable oil droplet geometry during the cooling process.

Figure 2 shows that there is a difference in contact angle between jatropha curcas oil, sunflower oil, and coconut oil, due to the different fatty acid compositions. The contact angles of the droplets changed differently with decreased temperature. This was due to the difference in viscosity of each vegetable oil, which affected the surface tension. The viscosity of each oil (measured using a Koehler capillary tube (ASTM D446)) is tabulated in Table 4.

Viscosity is influenced by the constituent fatty acids of vegetable oils. Jatropha curcas oil contains a large quantity of oleic acids and sunflower oil contains a large quantity of linoleic acids, while coconut oil contains a large quantity of lauric acids. Oleic acid (C_18_H_34_O_2_) is an unsaturated fatty acid with one double bond, linoleic acid (C_18_H_32_O_2_) is an unsaturated fatty acid with two double bonds, and lauric acid (C_12_H_24_O_2_) is a saturated fatty acid.

Differences in the structure of fatty acids caused differences in the London force. The dipole-generated interactions arising from the movement of electrons in oleic acid molecules are stronger than that of linoleic acid molecules. The interaction of linoleic acids is still stronger than that of lauric acids, and this stronger interaction causes higher viscosity. Therefore, jatropha curcas oil shows the highest droplet contact angle. Meanwhile, the contact angle of sunflower oil is slightly smaller, and the smallest droplet contact angle is of coconut oil.

The molecular structure of fatty acids also affects the polarity of fatty acids [15]. Oleic acid has weak polar properties (similar to linoleic acid), whereas lauric acid has a strong polarity. This polarity affects the water bonding force on the droplet surface, because a higher polarity will increase the water bonding force (as shown in Figure 2). After water vapor adhered to the droplet surface, the droplet color changed. In the jatropha curcas and sunflower oils the discoloration from light to dark was caused by the destructive interference of light rays, because water vapor adheres to the droplet surface. On another hand, a lighter color was seen in coconut oil after moisture adhered to the droplet surface. In coconut oil, it was found that moisture adhered throughout the droplet surface after cooling. In jatropha curcas and sunflower oils, the discoloration from light to dark occurred especially at the edges of the droplets.

**Figure 3:**
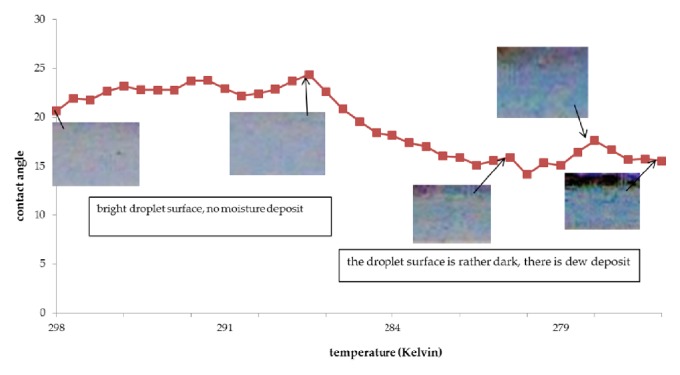
Contact angle of jatropha oil vs. temperature.

**Figure 4:**
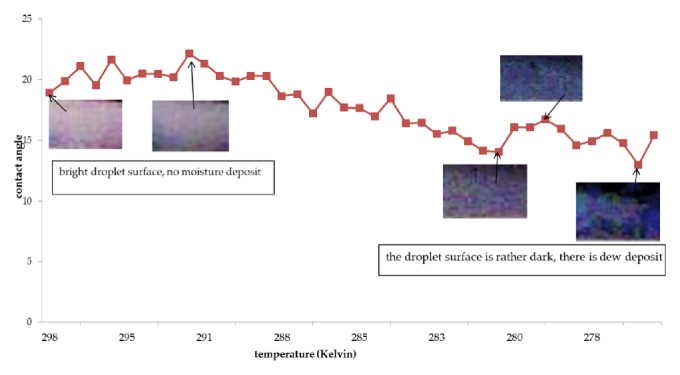
Sunflower oil contact angle vs. temperature.

**Figure 5:**
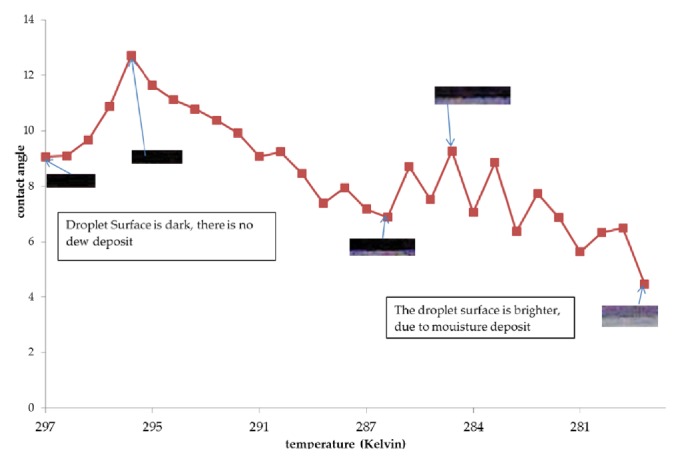
Coconut oil contact angle vs. temperature.

The results of the contact angle measurements are shown in the graphs of Figures 3, 4, and 5. The vertical axis shows the contact angle, and the horizontal axis shows the temperature. The temperature was varied from room temperature to approximately 273.15 K.

From the figures it is apparent that there are four stages in the cooling process: the first increases the contact angle, the second lowers the contact angle, the third again increases the contact angle, and the last lowers the contact angle again.

Figure 3 shows the contact angle and surface of the jatropha curcas oil droplet during the cooling process. The difference in color between the images of the jatropha curcas oil was caused by water adhering to the surface. Further, these different colors occurred because of the differences in refractive index between the oil and water, which is a symptom of light interference. As the light waves pass through the water layer on the surface of jatropha curcas oil, they experience a destructive interference, causing the moisture-filled areas to appear darker.

At room temperatures jatropha curcas oil droplets form a contact angle of 20.65°. At the initial cooling stage, the contact angle increased to 24.37° at 287.11 K. During this process the surface tension increased. At the second cooling stage, the contact angle reduced to 14.17° at 279.87 K. During this stage the surface tension decreased, due to water vapor condensation on the droplet surface. In the third cooling stage an accumulation of moisture formed on the droplet surface causing an apparent increase in contact angle to 17.65° at 277.43 K. During the final cooling stage the contact angle decreased, because the moisture could not be retained by the droplet.

The contact angle and surface color of a sunflower oil droplet are shown in Figure 4. The symptoms of destructive interference are clearly shown, whereby the sunflower oil color is brighter while the moisture color appears darker.

At the initial cooling stage, the contact angle of the sunflower oil droplet increased to 22.13° at 292.34 K. The maximum contact angle of the sunflower oil droplet was obtained at a higher temperature than the maximum droplet contact angle of jatropha curcas oil. In the second cooling stage, the contact angle decreased to 14.02° at 280.84 K. At this stage, the minimum contact angle was also smaller than the contact angle of the jatropha curcas oil droplet. Moreover, the droplet contact angle of sunflower oil was achieved at a higher temperature compared to jatropha curcas oil. At the third cooling stage, moisture accumulation occurred on the droplet surface, causing an apparent increase in contact angle to 16.69° at 279.27 K. During the final cooling stage the contact angle decreased, because the droplet could not withstand the moisture accumulation.

The contact angle and droplet surface color of coconut oil are shown in Figure 5. Based on the images in Figure 5, coconut oil appears dark. This dark color is because coconut oil tends to form a small contact angle, like a thin layer/film covering a glass substrate. Therefore, coconut oil also forms a destructive interference, causing it to appear dark.

At room temperature the coconut oil droplet formed a 9.06° contact angle. The coconut oil droplet contact angle was the smallest, when compared to jatropha curcas and sunflower oils. The contact angle increased to 12.7° at 295.59 K, due to an increase in surface tension during the initial cooling stage. The contact angle of coconut oil was reached at a higher temperature than both jatropha curcas and sunflower oils. The second cooling stage produced water vapor condensation on the droplet surface, causing a decreased contact angle of 6.88° at 286.46 K. During this stage the surface tension decreased. The third cooling stage resulted in moisture accumulation on the droplet surface, causing an apparent increase in contact angle to 9.27° at 284.29 K. At this stage the contact angle formed by coconut oil was less than for sunflower oil. Furthermore, during the final cooling stage the contact angle decreased, due to moisture accumulation that could not be retained by the droplet.

**Figure 6:**
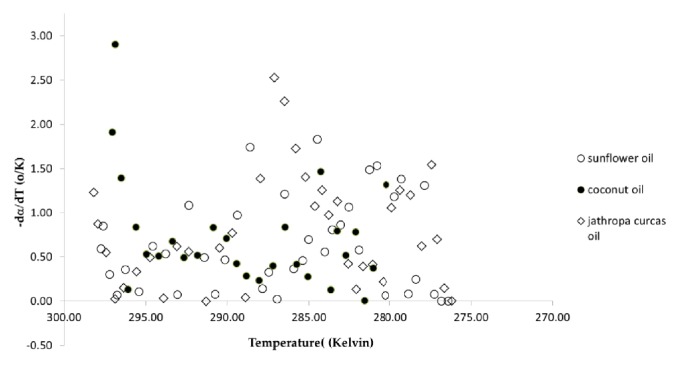
Slope of (-d*α*/dT) vs T.

The changes of contact angles of vegetable oil droplets (due to decreases in temperature (-d*α*/dT)) were affected by intermolecular forces. The relationship between (-d*α*/dT) and temperatures (T) is shown in Figure 6.

In the initial cooling stage coconut oil had a rapid change of contact angle with a temperature decrease from 300 to 295 K. Meanwhile, the contact angle change of sunflower oil was relatively fast during the cooling range from 288.15 to 284.15 K. For jatropha curcas oil, there was a rapid change of contact angle during cooling from 287.15 to 281.65 K.

Coconut oil exhibited a rapid change in contact angle at high temperatures, due to the intermolecular force in lauric acid. Lauric acid has a high degree of polarity, thus forming an intermolecular attraction between molecules, and this attractive force attracts surrounding molecules closer. A decrease in temperature causes molecules to vibrate at a lower frequency, which affects the high attractive speed of molecules.

Sunflower oil exhibited a rapid contact angle change when cooling from 288.15 to 284.15 K, while jatropha curcas oil exhibited a rapid contact angle change when cooling from 287.15 to 281.65 K. Sunflower oil contains linoleic acid, while jatropha curcas oil contains oleic acid, and both these acids are classified as nonpolar molecules. The attractive force between nonpolar molecules is caused by a temporary dipole, causing the force to be very weak. Therefore, these nonpolar molecules require a lower temperature than lauric acid to attract molecules, due to their weak attractive intermolecular force. A lower temperature will reduce the movement of the molecules and ease the attractive force due to the temporary dipole. Oleic acid is more nonpolar than linoleic acid; therefore the required temperature is also lower than linoleic acid.

## 4. Discussion

A contact angle is formed by surface tension, and the higher the surface tension, the greater the contact angle. Further, surface tension is influenced by intermolecular forces.

**Figure 7:**
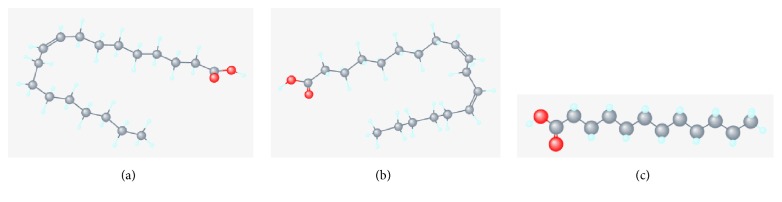
(a) Oleic acid [12], (b) linoleic acid [13], and (c) lauric acid [14].

Jatropha curcas oil is composed of oleic acid (41.29%) (the highest of the tested oils), linoleic acid (33.55%), and some other fatty acids. Sunflower oil contains linoleic acid (66.19%), oleic acid (22.35%), and other fatty acids. Meanwhile, coconut oil has a fatty acid composition of 40.77% lauric acid, 13.36% tetradecanoic acid, and other fatty acids. The molecule structure geometries of oleic, linoleic, and lauric acid (the main components of coconut, jatropha curcas, and sunflower oils) are presented in Figure 7.

The largest contact angle (*α*) at room temperature occurred in jatropha curcas oil (20.65°), followed by sunflower oil (18.92°) and coconut oil (9.06°). This is in accordance with Washburn's theory (cited by [16]) that high viscosity causes a large contact angle. According to [17], the viscosity is influenced by molecular size and form.

Oleic acid (C_18_H_34_O_2_) belongs to the group of long-chain molecules. Linoleic acid (C_18_H_32_O_2_) is a little shorter than oleic acid, while lauric acid (C_12_H_24_O_2_) is much shorter than oleic acid, as shown in Figure 7.

**Figure 8:**
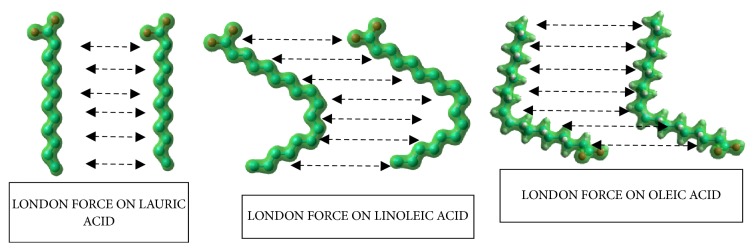
London force on lauric acid, linoleic acid, and oleic acid.

The left figure of Figure 8 shows a short fatty acid forming a droplet with a small contact angle, because it has a small London force. Meanwhile, the middle and right figures of Figure 8 show long fatty acids that comprise a droplet with larger contact angles. A longer chain will enlarge the electron cloud, and a larger electron cloud will enhance the London force. Furthermore, an improved London force will increase the viscosity [18].

Decreased temperatures increased the contact angles of all types of vegetable oils tested. Jatropha curcas oil contact angle increased from 20.65° to 24.37°; sunflower oil increased from 18.92° to 22.13°; coconut oil increased from 9.06° to 12.7°. Decreased temperatures will increase the viscosity of the liquid, which in turn increases the surface tension. Moreover, the contact angle will be greater due to the increased surface tension [19]. A decrease in temperature causes a decrease in the molecular kinetic energy (molecular vibrations), which causes a decrease in the distance between atoms. This reduced distance between molecules results in an increased interaction force between the molecules; therefore the surface tension and contact angles also increase.

**Figure 9:**
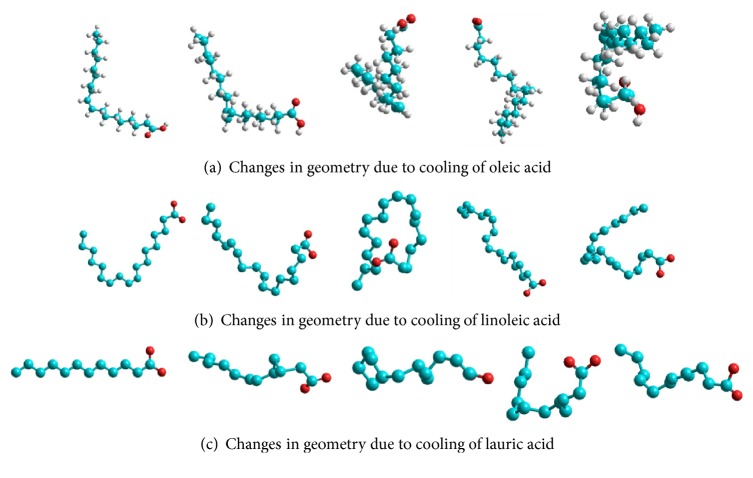


Based on simulations using chemistry software, during cooling there was a decrease of kinetic energy visualized with molecular geometry changes, as shown in Figure 9.

It can be seen in Figure 9 that the cooling process causes the distance between atoms in the molecules to become closer, indicating that the vibrations of the atoms weakened (or the kinetic energy decreased).

**Figure 10:**
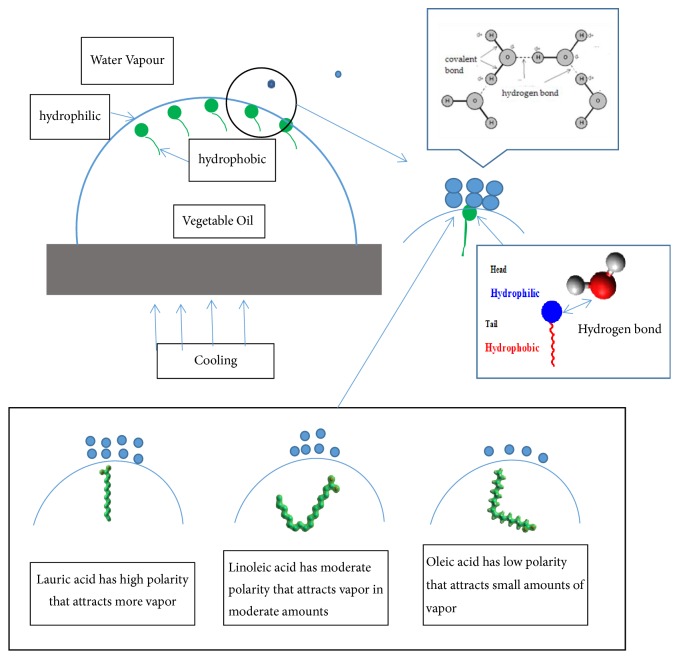
Intermolecular forces for moisture attraction on vegetable oil droplet surfaces.

Figure 10 illustrates that fatty acids are molecules with hydrophilic properties on the head and hydrophobic parts of the tail. The hydrophilic head attracts moisture around it, and when the temperature decreases there is a condensation of vapor to water. The water is then attracted to the droplet surface of the vegetable oil [19].

The formation of deposits on the droplet surface is influenced by the hydrophilic properties. Lauric acid has a highly hydrophilic nature and therefore attracts more surface water vapor than both linoleic and oleic acids. Consequently, the contact angle change of coconut oil is greater than that of sunflower and jatropha curcas oil.

Decreased temperature decreases the movement of water vapor molecules in air. A slight movement indicates a small amount of kinetic energy, and because the kinetic energy is small the intermolecular forces attract adjacent molecules. After bonding, water vapor molecules turn into a liquid and will turn into ice if cooled continuously [20].

**Figure 11:**
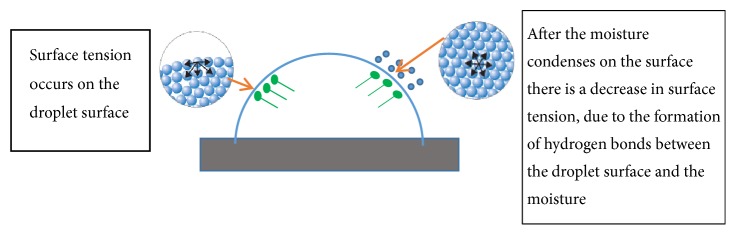
The formation and decrease of surface tension.

The hydrogen bonds that occur between the molecules on the droplet surface (and moisture adhered to the droplet surface) decrease the surface tension (see Figure 11). A decreased surface tension will result in a decreased droplet contact angle in vegetable oils. Based on the experimental results, the decrease in contact angle of jatropha curcas oil was from 24.37° to 14.17°. The contact angle of sunflower oil reduced from 22.13° to 14.02°, and that of coconut oil reduced from 12.7° to 6.88°.

After the contact angles decreased, they appeared to increase again. The increased droplet contact angle of jatropha curcas oil was from 14.17° to 17.65°. Meanwhile, the droplet contact angle of sunflower oil increased from 14.02° to 16.69°, and that of coconut oil increased from 6.88° to 9.27°. This increase was due to the accumulation of moisture condensed on the droplet surface, and water molecules bind each other to hydrogen bonds (see Figure 11).

Hydrogen bonding occurs due to the attraction (interaction) between dipoles of hydrogen atoms and oxygen. An attractive force occurs because oxygen has high electronegativity, and hydrogen is a nonmetallic element that has low electronegativity. When hydrogen binds to highly electronegative elements (such as oxygen), the combined electron couples are more attracted to oxygen, so that oxygen becomes more negative than hydrogen [21].

In the condensation process on the droplet surface, the distance between water molecules is close enough for attraction, resulting in water accumulation [22]. This excess accumulation of water molecules presses the droplet surface; therefore the contact angle will again decrease.

This research found that the droplet contact angle of jatropha curcas oil decreased from 17.65° to 15.5°. Further, the droplet contact angle of sunflower oil decreased from 16.69° to 12.94°, while that of coconut oil reduced from 9.27° to 4.46°.

## 5. Conclusions

This research has validated that changes in contact angles of vegetable oil droplets due to decreased temperatures are influenced by the intermolecular forces in their constituent molecules. Further, increased contact angles occur due to the decreased kinetic energy of molecules, and this decrease in kinetic energy is evidenced by changes in molecular geometry.

One of these intermolecular forces is London force, and its interaction increases as the temperature decreases. This results in an increase in the viscosity of the liquid, which changes the contact angle.

The hydrophilic properties of fatty acid molecule heads attract moisture to the droplet surface, and the water vapor adhered to the droplet surface forms hydrogen bonds.

Hydrogen bonds contribute to contact angle changes, and they are formed by the high electronegativity in oxygen with the more positive hydrogen. Hydrogen bonds bind water to the droplet surface and this damages the surface tension, thus lowering the contact angle on the droplet.

Moreover, hydrogen bonds bind water in greater amounts; therefore, water is accumulated on the droplet surface. Water accumulation causes the contact angle to seemingly increase. In large quantities, water accumulation will depress the droplet, thereby decreasing the contact angle.

## Figures and Tables

**Table 1:** Fatty acid composition of coconut oil.

Fatty Acid	Butyric acid	Octanoic Acid	Decanoic acid	Lauric acid	Tetradecanoic acid	Palmitic acid	Oleic acid
Percentage	6,73	6,44	5,62	40,77	13,36	11,27	10,85

**Table 2:** Fatty acid composition of Jatropha curcas oil.

Fatty Acid	Octadecanoic acid	Oleic Acid	Linoleic acid	Eicosenoic acid
Percentage	9,9	41,29	33,55	3,53

**Table 3:** Fatty acid composition of sunflower oil.

Fatty Acid	Palmitic acid	Elaidic acid	Oleic Acid	Linoleic Acid
Percentage	6,27	2,96	22,35	66,19

**Table 4:** Viscosity at room temperature.

Type of oil	Kinematic Viscosity (Ns/m^2^)
Jatropha curcas oil	0,0199
Sunflower oil	0,005525
Coconut oil	0,0033

## Data Availability

The data used to support the findings of this study are available from the corresponding author upon request.
